# Neuronal Categorization and Discrimination of Social Behaviors in Primate Prefrontal Cortex

**DOI:** 10.1371/journal.pone.0052610

**Published:** 2012-12-28

**Authors:** Joji Tsunada, Toshiyuki Sawaguchi

**Affiliations:** 1 Laboratory of Cognitive Neurobiology, Hokkaido University Graduate School of Medicine, Sapporo, Hokkaido, Japan; 2 Humanity Neuroscience Institute, Sapporo, Hokkaido, Japan; 3 Musashino Gakuin University, Sayama, Saitama, Japan; Roma Tre University, Italy

## Abstract

It has been implied that primates have an ability to categorize social behaviors between other individuals for the execution of adequate social-interactions. Since the lateral prefrontal cortex (LPFC) is involved in both the categorization and the processing of social information, the primate LPFC may be involved in the categorization of social behaviors. To test this hypothesis, we examined neuronal activity in the LPFC of monkeys during presentations of two types of movies of social behaviors (grooming, mounting) and movies of plural monkeys without any eye- or body-contacts between them (no-contacts movies). Although the monkeys were not required to categorize and discriminate the movies in this task, a subset of neurons sampled from the LPFC showed a significantly different activity during the presentation of a specific type of social behaviors in comparison with the others. These neurons categorized social behaviors at the population level and, at the individual neuron level, the majority of the neurons discriminated each movie within the same category of social behaviors. Our findings suggest that a fraction of LPFC neurons process categorical and discriminative information of social behaviors, thereby contributing to the adaptation to social environments.

## Introduction

It has been reported that primates show excellent social-cognition and execute appropriate social-interactions with other individuals depending on social behaviors among them [1], [2], [3], [4], [5], [6]: e.g., challenges of taking a female away by a rival male change depending on whether a male has strong grooming relationships with the female or not [7], [8]. These findings suggest that primates have an ability to know what kind of social behaviors other individuals are doing (i.e., categorize social behaviors between other individuals), but neuronal mechanisms for such a categorization of social behaviors are poorly understood.

Recent studies suggest that several brain regions are involved in the processing of social information and/or the production of emotional/social behaviors (e.g., the anterior cingulate cortex [9], [10], [11]; the insula [12]; the lateral intraparietal area [13], [14]; the amygdala [15], [16], [17]). Among the brain regions processing social information, the lateral prefrontal cortex (LPFC) is likely to be involved in the categorization of social behaviors as follows. First, the LPFC plays a central role in the categorization of various kinds of stimuli [18], [19], [20], [21], [22], [23]. Second, the LPFC has been implicated in the processing of social stimuli. For example, neurons in the monkey LPFC process faces/vocalizations [24], [25], [26], [27], [28], [29], [30], [31], [32], [33], [34] and modulate their activities depending on social contexts [35]. In addition, the LPFC receives direct projections from the inferotemporal cortex, the superior temporal sulcus, and the superior temporal gyrus which are involved in the processing of faces and vocalizations [36], [37], [38], [39]. Moreover, lesions of the LPFC induced deficits in appropriate social-interactions [40], [41], [42], [43], and these impairments appear to be caused by not only failures to execute social-interactions, but also failures to process, including categorize, social behaviors among other individuals. Also, in humans, damage to the LPFC induced impairments of processing visual information of social behaviors and facial expressions [44], [45]. Finally, in the nonhuman primate, the positive correlation between volumes of grey matter in the LPFC and numbers of members living together implies the importance of the LPFC in the social life [46].

**Figure 1:**
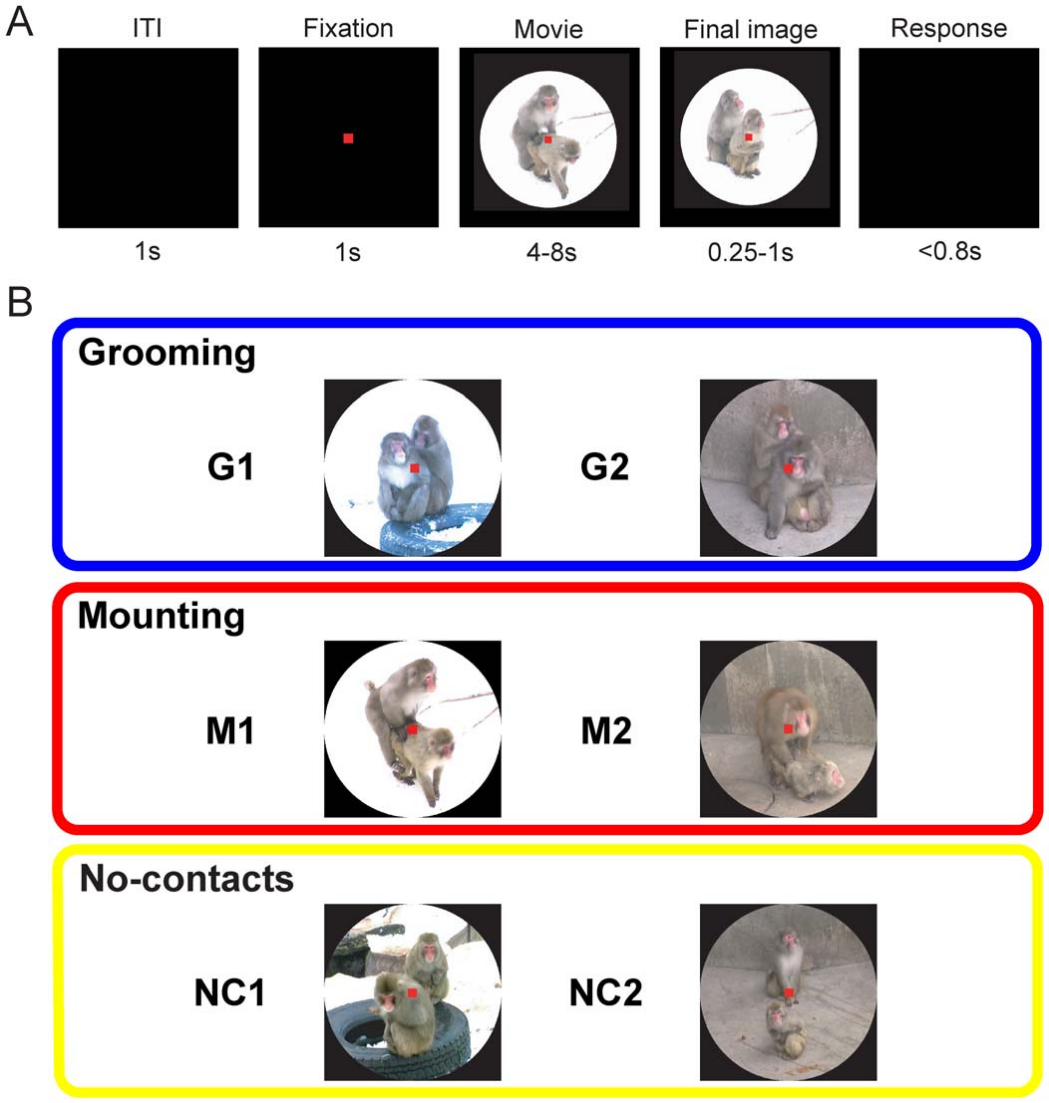
Behavioral task and movie stimuli. (A) Temporal sequence of our reaction time task. (B) Clips of three types of movie stimuli in the standard set (top: two examples of grooming movies, G1, G2; middle: mounting movies, M1, M2; bottom: no-contacts movies, NC1, NC2).

Thus, it appears that primates have an ability to categorize social behaviors, and the LPFC plays a key role for the ability. We therefore hypothesized that the primate LPFC is involved in the categorization of social behaviors. To address this hypothesis, we examined neuronal activity in the LPFC of monkeys during presentations of two types of movies of social behaviors (grooming, mounting) and movies of plural monkeys without any eye- or body-contacts (no-contacts movie) (see Fig. 1). We report here that a fraction of LPFC neurons modulated their activities by the type of social behaviors even when the monkeys were not required to categorize and discriminate the movies, suggesting an involvement of the LPFC in the categorization and discrimination of social behaviors.

## Materials and Methods

### General Procedures

Two macaque monkeys (*Macaca fuscata*, about 13kg and about 8kg, named MK and KS, respectively) were used as subjects. All procedures were approved by the Animal Care and Use Committee of Hokkaido University School of Medicine (Protocol Number: 06034). In addition, the subjects were treated in accordance with “The Guide for the Care and Use of Laboratory Animals” and “The use of non-human primates in research”. According to the guidelines, we made all efforts for primate care and welfare. For example, the monkeys were kept in individual primate cages in an air-conditioned room. Their health condition (e.g., body weight, behavior, and appetite) was checked daily. Supplementary fruits were provided daily.

Before training, the monkeys were habituated to a monkey chair and then a head holder was implanted by aseptic surgery under pentobarbital anesthesia (about 25 mg/kg, i.v.), as described in our previous studies [47], [48]. Prophylactic antibiotics were injected i.m. on the day of surgery and daily for a week after surgery. After recovery from the surgery, the monkeys were trained to sit on a primate chair inside a dark room with head fixation.

### Behavioral Task and Movie Stimuli

The monkeys were trained to perform a reaction time task (Fig. 1A). This task started when the monkey pushed a lever located at waist level and fixated on a central fixation point (a red square, 0.5° × 0.5°) on a CRT monitor. After 1s, a movie (10° × 10°) was presented at center (movie period) of the CRT. Following the presentation of the movie, the final image of the movie lasted from 0.25 s to 1 s (final image period). Then the image was turned off, which cued the monkey to release the lever. When the monkey released the lever within 800 ms from the offset of the final image presentation, a drop of water (the duration of reward delivery: 75 ms) was delivered into the monkey’s mouth (reward period). For each trial, a movie was selected pseudo-randomly (i.e., frequencies of presentations of each movie were same). We defined a reaction time as a time between the offset of the final image presentation and the lever release. The final image period was introduced to randomize the timing of the period for the lever release, thereby preventing the monkeys from estimating the timing of the lever release without paying attention to the movies. Throughout the trial, eye positions were restricted within the 2.5° window from the fixation point. We selected this window size because most of the monkeys’ behaviors in the movies fell within the 2.5° window. If eye positions fell out the window, the trial was aborted, being restarted after the inter-trial interval (1 s).

The task and recordings were controlled by a system that consisted of an infrared eye-camera system (R-21C-A; RMS, Hirosaki, Japan), three personal computers, and other associated peripheral equipments. The eye-camera system was connected to the personal computers via A/D converters and was used for monitoring and sampling eye positions. The three personal computers were networked by RS232C and parallel I/O. Two of the computers controlled the task, while another monitored and collected the data for the neuronal activity, eye positions, and task events.

To examine effects of the type of social behaviors, we prepared three types of silent movies (Fig. 1B and Video S1, S2, S3): grooming (manipulation of the skin or fur of another by hands or mouth), mounting (mounting and thrusting another from behind), and no-contacts (no eye- and body-contacts between individuals). Each movie contained female and male monkeys, which were unfamiliar to the subjects, being filmed (25 frames/s) at Maruyama zoo in Sapporo, Japan.

To analyze the neuronal activity adequately, we used two sets of movies: a standard set (2 movies of each type, 6 movies total) and a confirmation set (10 movies). Since grooming in the field is conducted by one individual (self-grooming) or more than two individuals (social-grooming), we prepared movies of grooming conducted with more than two individuals (social-grooming) and movies of plural monkeys’ grooming conducted individually (self-grooming) in the confirmation set (see each type of grooming in the Video S1). Furthermore, to test the possible influence of small/fractural movements of faces/bodies, rather than an overall pattern of movements of social behaviors, on the neuronal activity, we prepared mosaic movies and scrambled movies of one of each type of the movies in the standard set (3 mosaic movies and 3 scrambled movies; see Figure S1, and Video S1, S2, S3). To make mosaic movies, we used a mosaic-effect tool of video editing software Ulead Media Studio Pro (i.e., cutting each frame into 1156 (34 × 34) rectangles and pixelizing each rectangle). Scrambled movies were made by cutting each frame into 64 (8 × 8) rectangles and scrambling positions of the rectangles. In the mosaic stimuli, small/local movements (e.g., facial expressions, movements of fingers) in the original movies were removed, whereas the overall pattern of movements was preserved (see Figure S1). In the scrambled stimuli, small/local movements were preserved, whereas the overall pattern of movements was removed (see Figure S1). Therefore, these mosaic and scrambled stimuli were useful for the examination of the effects of small/local movements of faces/bodies and the overall pattern of movements of social behaviors. We used either mosaic or scrambled stimuli (control set) in each recording session. The monkeys were usually tested with the standard and control sets, and in some recording sessions, we added the confirmation set. The monkeys daily performed ∼600 trials of the task with the standard set of the movies and therefore each movie was presented ∼66 times on average.

For the standard set of movies, to examine the effect of the movie contents (e.g., monkeys’ movements and appearance of their faces) on the neuronal activity more closely, we made lists of the contents of the movies (Figure S2). For the grooming movies, we listed time points of grooming with the hands and those of grooming with the mouth. In the grooming movies, the monkeys kept grooming from the start to the end of the movies. For the mounting movies, we listed time points that 1) the male pushed the female, 2) the female touched the ground, 3) the double foot clasp position started (i.e., the male monkey places his hands flat on the female monkey’s sacrum and clasps her hind limbs with his feet [49]; see also movie clips of M1 and M2 movies in Fig. 1B), 4) the male thrusted, 5) the male released the clasping of the female’s hind limbs, and 6) the female released her hands from the ground. Also we listed appearances of the monkeys’ faces. On a frame by frame basis, we scored the appearance of the face as 0, 0.5, or 1. If the face of a monkey was entirely observed, we scored it as 1. If the face was overlapped with another monkey’s body or the monkey showed the side face (i.e., only one eye was observed), we scored it as 0.5. If most parts of the face were not observed, we scored it as 0. Mean face appearance scores for each movie was: G1 male (female): 1.00 (0.77), G2 male (female): 1.00 (0.69), M1 male (female): 0.69 (0.78), M2 male (female): 0.63 (0.74), NC1 male (female): 0.95 (0.93), NC2 male (female): 1.00 (0.89).

### Recording Procedures

After the training was completed, implantation of a recording cylinder (20 × 40 mm) was performed by aseptic surgery under pentobarbital anesthesia (about 25 mg/kg, i.v.), as described previously [47], [48]. Prophylactic antibiotics were injected i.m. on the day of surgery and daily for a week afterwards. The activity of single neurons was recorded with custom-made glass-insulated elgiloy microelectrodes (0.5-1 MΩ), using conventional electrophysiological techniques similar to those described in our previous studies [47], [48]. The microelectrode was positioned using a pulse motor-driven micromanipulator (MO-81; Narishige, Tokyo, Japan) and plastic grid with numerous small holes (0.7 mm internal diameter, 1.5 mm apart from each other). We advanced the electrode until the activity of one or more neurons was well-isolated and then commenced data recording. Data for the neuronal activity were digitized by Multi-Spike Detector (Alpha Omega Engineering, Nazareth, Israel). These digitized data were stored in a data-collection computer and analyzed off-line. Furthermore, all the analogue data (i.e., neuronal activity, eye position, and task sequence) were recorded on digital audiotape (DAT) using an eight-channel DAT recorder (PC208M; Sony, Tokyo, Japan).

**Figure 2:**
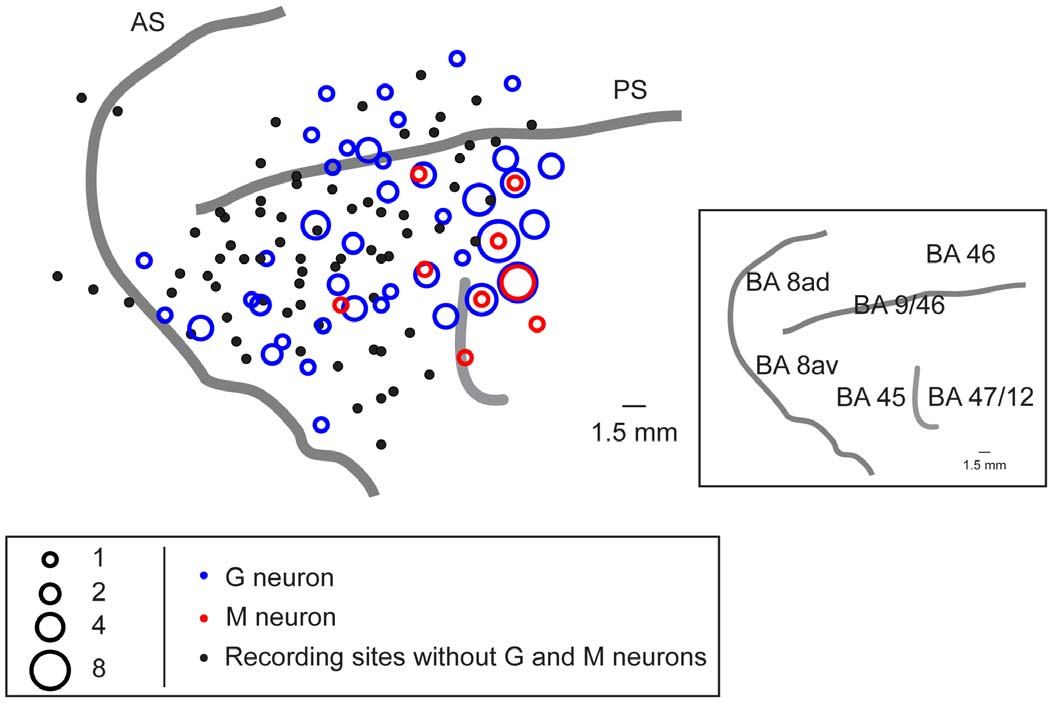
Recording sites of G and M neurons, which are illustrated on the cortical surface of the LPFC. The sizes of the blue, red circles indicate the numbers of G and M neurons recorded at each site, respectively. Black dot, recording sites without G and M neurons. The right inset shows a diagram of brain areas [39]. AS, arcuate sulcus. PS, principal sulcus. BA, Brodmann area.

We focused on neurons in the LPFC rostrolateral to the frontal eye field (FEF) (Fig. 2). To estimate the FEF physiologically, we applied the intracortical microstimulation (ICMS; 22 cathodal pulses of 0.3 ms duration at 333 Hz, up to 100 µA) through the recording electrode. When eye movements were elicited by the ICMS, the site was considered to be within the FEF [50], and data recorded from these sites were excluded.

### Data Analyses

For statistical tests, instead of making the assumption of equal variances for testing samples, we used *t*-test and ANOVA with Welch’s correction for unequal variances. First, to examine whether a neuron showed a significant modulation in the activity during the movie period, we compared the firing rate of the neuron during the whole movie period (e.g., in Fig. 1B, G1∶7.16 s, G2∶6.36 s, M1∶7.56 s, M2∶6.84 s, NC1∶7.24 s, NC2∶7.16 s) and that during the fixation period (*t*-test, *P*<0.05). If a neuron showed a significantly different activity, we defined the neuron as a movie-responsive neuron. In the following analysis, we focused on the movie-responsive neuron. Since we were primarily interested in the effects of the type of social behaviors on the neuronal activity, we applied a one-way ANOVA to the neuronal activity during the whole movie period (the type of social behaviors as a factor) (*P*<0.05) and post-hoc comparison with Tukey-Kramer test (*P*<0.05). Neurons with a significantly different activity in a specific type of social behaviors in comparison with the others involved two types: a “Grooming neuron (G neuron)”, which showed a significantly different activity in grooming compared to no-contacts and mounting and a “Mounting neuron (M neuron)”, which showed a significantly different activity in mounting compared to no-contacts and grooming. Neurons which showed significantly different activities in both grooming and mounting compared to no-contacts were not included in G and M neurons. In this study, we focused on G and M neurons because these neurons may code preferentially visual information of grooming and mounting, respectively.

The activity of some of these G and M neurons were examined with the mosaic or scrambled movies. In these examinations, we applied a two-way ANOVA to examine effects of the movie content (i.e., grooming, mounting, and no-contacts) and the movie type (i.e., original vs. mosaic or scrambled) (*P*<0.05), and performed a post-hoc comparison with Tukey-Kramer test (*P*<0.05). In addition, the activity of some G neurons was further examined with the movies of self-grooming of plural monkeys, social-grooming, and no-contacts; in this examination, we applied a one-way ANOVA (the type of the movies as a factor) (*P*<0.05) and a post-hoc comparison with Tukey-Kramer test (*P*<0.05).

During the present examination, we had recognized that G and M neurons might show categorical and/or discriminative activities for different movies (see Results section) and, hence, we performed quantitative analyses for the categorization and discrimination. As for the neuronal categorization of social behaviors, we first divided the movies into two categories: for the G neuron, grooming category and non-grooming (i.e., mounting and no-contacts) category; for the M neuron, mounting category and non-mounting (i.e., grooming and no-contacts) category. Then, to evaluate whether individual neurons responded more similarly to movies within the same category than between different categories, we computed two parameters: an average within-category difference (WCD) in the firing rate between pairs of movies in the same category and an average between-category difference (BCD) in the firing rate between pairs of movies from different categories [19], [20]. Therefore, for the standard set stimuli, WCD was the average of absolute differences in the activity of 7 pairs (e.g., for G neuron, differences between G1 and G2, M1 and M2, NC1 and NC2, M1 and NC1, M2 and NC2, M1 and NC2, and M2 and NC1) and BCD was the average of absolute differences in the activity of 8 pairs (e.g., for G neuron, differences between G1 and M1, G1 and M2, G1 and NC1, G1 and NC2, G2 and M1, G2 and M2, G2 and NC1, and G2 and NC2). Furthermore, to measure the strength of the neuronal categorization of social behaviors, we calculated a social-behavior categorization index (SCI), as follows: SCI = (BCD - WCD)/(BCD+WCD). Values of the index ranged from -1 to 1. Positive values of the index indicate larger differences for movies in different categories and negative values larger differences within each category.

For the quantitative analysis of the neuronal discrimination of each movie within categories, we examined how many neurons showed a significantly different activity across different movies within the same category in the standard set. In this examination, for example in G neurons, we applied a *t*-test (*P*<0.05) on the activity for grooming category movies (i.e., two movies) and a one-way ANOVA (*P*<0.05) on the activity for non-grooming category movies (four movies). According to this analysis, neurons were classified into two types: “categorical” neurons that did not show a significantly different activity within the same category of stimuli and “discriminative” neurons that showed a significantly different activity among stimuli of the same category. To examine the degree of the neuronal categorization of these two sub-types of neurons, respectively, we calculated SCIs, as described above.

### Estimation of Recording Sites

After recordings were completed, the monkeys were deeply anesthetized with an overdose of sodium pentobarbital and perfused with 0.9% saline, followed by 10% formalin. Then, to estimate the recording sites, we 1) positioned ∼5 microelectrodes in the recording sites by using the recording set-up (i.e., the micromanipulator and the grid), 2) removed the brain, and 3) photographed it. By using the electrodes as landmarks, we estimated the recording sites on the cortical surface. The distributions of the recorded neurons are illustrated in Fig. 2.

## Results

### Behavioral Performance

Throughout the recording sessions (8 months for monkey MK, 6 months for monkey KS), the monkeys performed the reaction time task with >95% correct responses for all the movies. On a session by session basis, we compared the reaction time for each movie by applying a one-way ANOVA (*P*<0.05). We didn’t find a significant difference in the reaction time in most of the recording sessions (360 of 362 sessions, 99.4%). Thus, a significant difference in the behavioral performance across the movies was not observed.

### Neuronal Classifications and Distributions

We recorded the activity of a total of 546 neurons in the LPFC (395 neurons from right hemisphere of monkey MK and 151 neurons from both hemispheres of monkey KS) during the presentations of the standard set of movies with two types of social behaviors (grooming, mounting) and no-contacts (see Fig. 1). Of the recorded neurons, 408 neurons showed a significantly different activity in the movie period compared to the fixation period (*t*-test, *P*<0.05). Of these 408 neurons, 142 neurons (35%) showed a significantly different activity for a specific type of social behaviors compared to the others in the movie period. These neurons involved G neurons (n  = 126; monkey MK: n  = 89; monkey KS: n = 37) and M neurons (n  = 16; monkey MK: n = 13; monkey KS: n = 3). The G-neuron was found frequently, and its proportion (126/142, 89%) was significantly larger than M neuron’s proportion (n  = 16/142, 11%) (χ^2^ test, *P*<0.05). These G and M neurons were distributed broadly within the recording sites of the LPFC including area 45, area 9/46, and area 47/12 [39], and we did not find obvious differences in the spatial distribution between G and M neurons (Fig. 2).

### Properties of G and M Neurons

**Figure 3:**
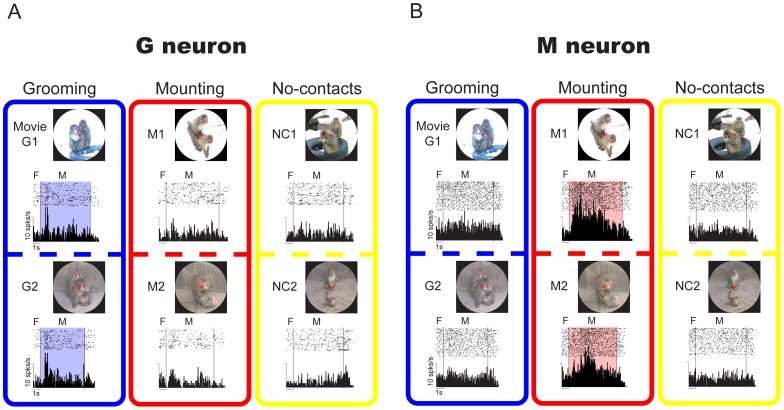
Examples of G and M neurons. (A) An example of a G neuron in which the activity in grooming movies was significantly different in comparison with the others. Movie clips, raster displays and averaged histograms for neuronal activity are shown according to the type of social behaviors. F, fixation period. M, movie period. (B) An example of an M neuron in which the activity in mounting movies was significantly different in comparison with the others. The format and abbreviations are the same as those in Fig. 3A.

An example of a G neuron is shown in Fig. 3A. In this figure, raster displays and averaged histograms of the neuronal activity are illustrated for each movie. In comparison with the activity during the period of no-contacts movies, this neuron showed a phasic increase in the activity during the period of grooming movies (one-way ANOVA and post-hoc comparison with Tukey-Kramer test, *P*<0.05), but showed little change in activity during the period of mounting movies (*P*>0.05). Figure 3B shows an M neuron. This neuron showed an increase in the activity during the period of mounting movies (*P*<0.05), but showed little change in the activity during the period of grooming movies (*P*>0.05). Like these examples in Fig. 3, all G and M neurons showed a significantly different activity in both the two grooming-movies and two mounting-movies, respectively, compared to the other movies.

**Figure 4:**
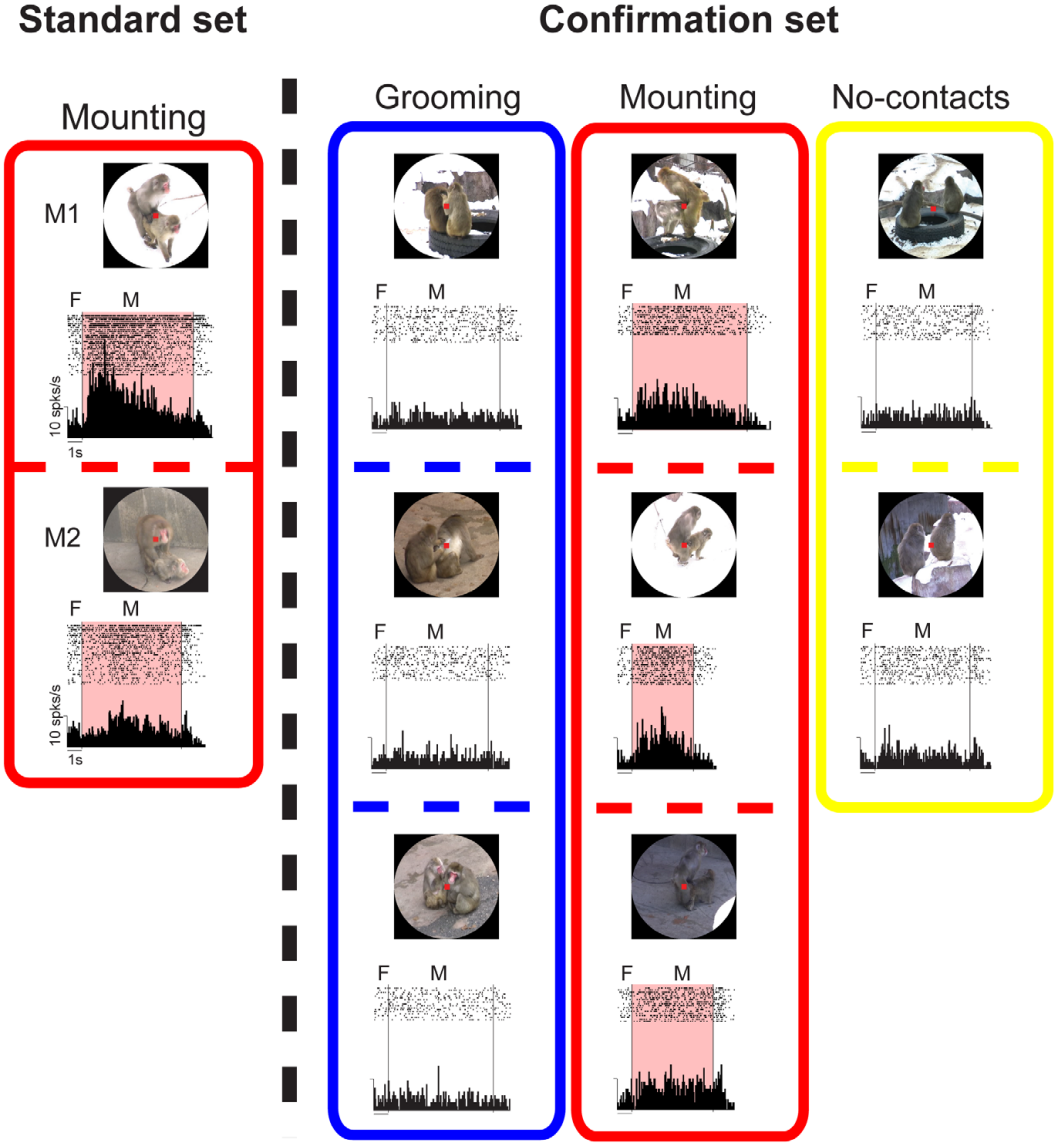
An example of an M neuron tested with the standard set and the confirmation set of movies. The format and abbreviations are the same as those in Fig. 3A.

As we usually examined the neuronal activity with the “standard” movie set (see Materials and Methods), G and M neurons might respond to only these familiar stimuli. To examine this possibility, a subset of G and M neurons were tested with different movies with similar social contents; i.e., the “confirmation” set of movies (see Materials and Methods). The majority of G (17/24, 71%) and M neurons (3/3, 100%), which were examined in both the standard and confirmation sets, showed a significant modulation of the activity in the different set of movies of grooming and mounting, respectively, compared to the other movies (one-way ANOVA and post-hoc comparison with Tukey-Kramer test, *P*<0.05) (Table 1), as evident in an M neuron shown in Fig. 4. This M neuron showed an increase in the activity in the mounting movies of both the standard set (*P*<0.05) and confirmation set (*P*<0.05) in comparison with the other movies. In contrast, this neuron showed little change in the activity during the period of grooming movies in the confirmation set. Thus, the familiarity of the stimuli was not critical for the activity of G and M neurons.

**Table 1:** The number of G and M neurons with a significantly different activity in the standard set of movies that showed a significantly/not-significantly different activity in the other set of movies.

	G neuron (n = 126^a^)			M neuron (n = 16^b^)		
	Significant	Not-significant	Total	Significant	Not-significant	Total
Confirmation movies	17	6^c^	24	3	0	3
Mosaic movies	96	25^d^	121	12	2^e^	14
Scrambled movies	0	5	5	0	2	2
Self-grooming movies	2^f^	15	17	–	–	–
Number of neurons usedfor populationanalyses	a-(either c or d or f) = 94			b-e = 14		

**Figure 5:**
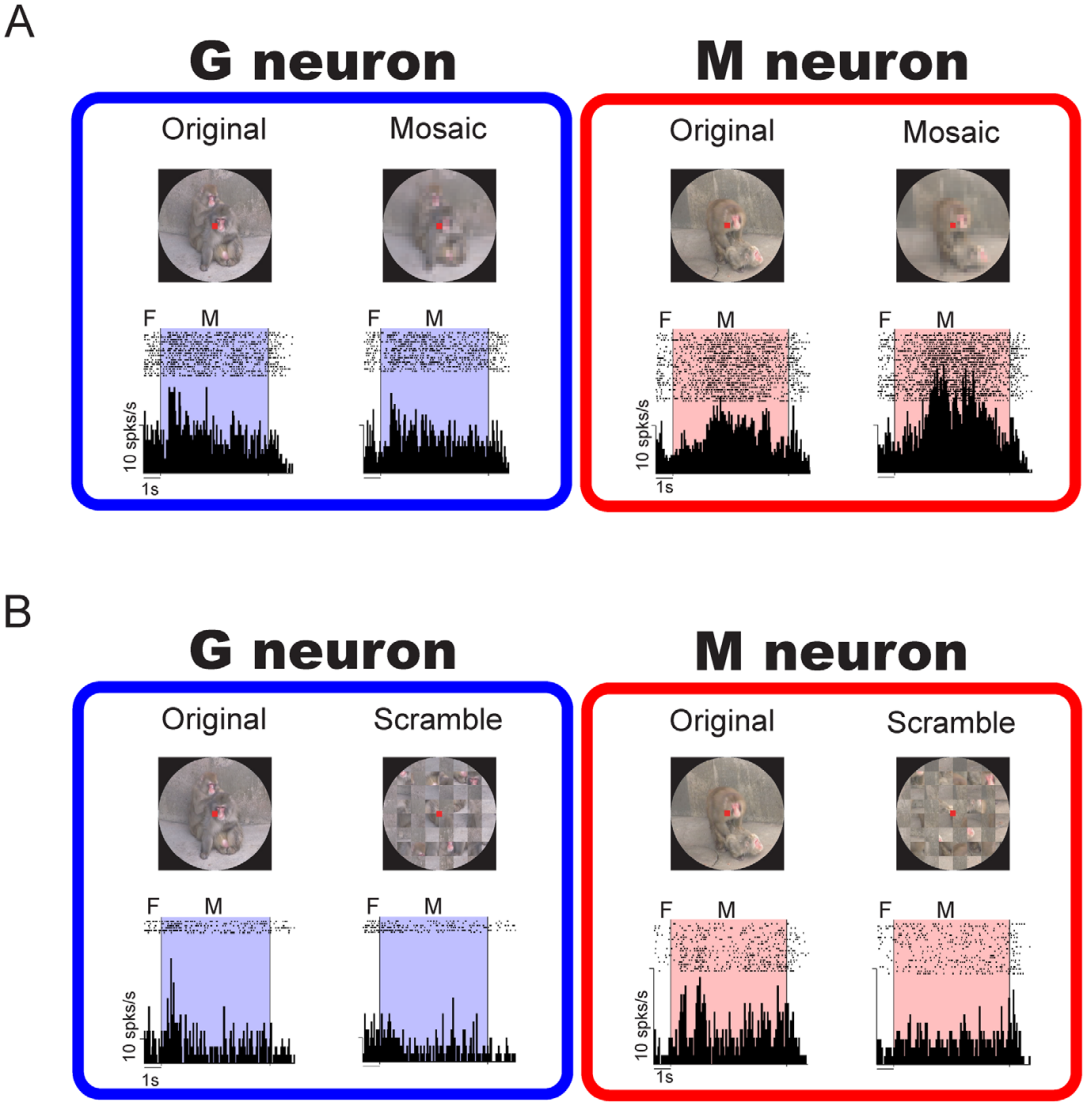
Examples of G and M neurons’ responses to mosaic movies and scrambled movies. (A) Examples of the activity of G and M neurons for original and mosaic stimuli. (B) Examples of the activity of G and M neurons for original and scrambled stimuli. The format and abbreviations are the same as those in Fig. 3A.

The social behaviors contained small/local movements of faces/bodies, and these movements, rather than the overall pattern of movements of social behaviors, might be critical for the activity of G and M neurons. To examine this possibility, we compared the neuronal activity for the original movies with that for the mosaic movies or that for the scrambled movies (see Materials and Methods, Figure S1, and Video S1, S2, S3). In these examinations, we applied a two-way ANOVA to examine the effects of the movie content (i.e., grooming, mounting, and no-contacts) and the movie type (i.e., original vs. mosaic or scrambled) (*P*<0.05). The majority of G (96/121, 79%) and M neurons (12/14, 86%) showed a significant main effect in the movie content, and post-hoc comparison with Tukey-Kramer test (*P*<0.05) revealed that those G and M neurons showed a significantly different activity in grooming and mounting movies, respectively, in comparison with the other movies even in the mosaic stimulus (Table 1) as examples shown in Fig. 5A. These G and M neurons showed similar activities in both the mosaic and original movies. In the scrambled movies, none of the G (5/5, 100%) and M neurons (2/2, 100%) showed a significant main effect in the movie content (Table 1) as examples of G and M neurons shown in Fig. 5B. These neurons did not show similar activities between the original and scrambled movies; the activity-modulation in the original movie decreased in the scrambled stimulus. These findings indicate that the differential activity of most of the G and M neurons during social movies is attributable to the overall pattern of movements of social behaviors rather than to small/local movements of faces/bodies.

G neurons were the most frequently found here, and grooming in the field is conducted by one individual (self-grooming) or more than two individuals (social-grooming) (see Video S1). We compared responses of G neurons during the confirmation set of movies with self-grooming of plural monkeys, social-grooming, and no-contacts (one-way ANOVA and post-hoc comparison with Tukey-Kramer test, *P*<0.05) (Table 1). In comparison with the no-contacts, most G neurons (15/17, 88%) showed a significantly different activity for the social-grooming, but not for the self-grooming. The remaining small number of G neurons (2/17, 12%) showed a significantly different activity for both self- and social-grooming.

### Population Activities of G and M Neurons

To examine the overall behavior of G and M neurons, we calculated their population activities as follows. For each neuron, the neuronal activity for each movie or movie type in the standard set was normalized (divided) by the neuronal activity during the fixation period. Next normalized neuronal activities were averaged across neurons. In this analysis, we excluded a subset of G and M neurons that did not show significant modulations in either the confirmation set of movies or the set of the mosaic movies (Table 1). We also excluded the small number of G neurons that showed a significant modulation in self-grooming, and thus analyzed 94 G neurons and 14 M neurons for the standard set and 15 G neurons and 3 M neurons for the confirmation set.

**Figure 6:**
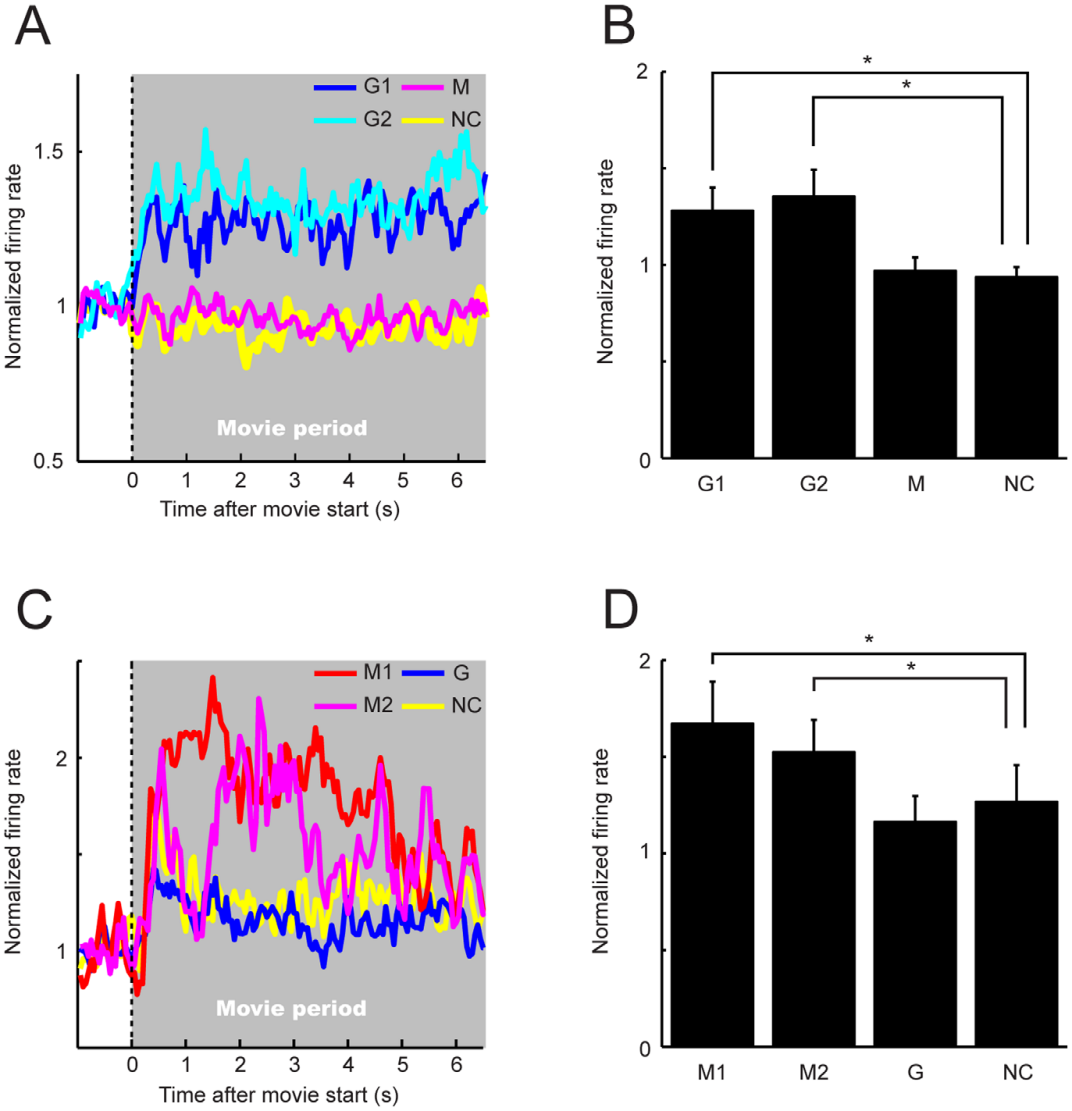
Population normalized-activities of G and M neurons. Population histograms of (A) G (n  = 94) and (C) M (n  = 14) neurons were aligned at the movie start. G1 (G2), the activity for the G1 (G2) movie. G, the averaged activity for two grooming-movies. M1 (M2), the activity for the M1 (M2) movie. M, the averaged activity for two mounting-movies. NC, the averaged activity for two no-contacts-movies. Mean normalized activities (mean ± S.E.) of (B) G and (D) M neurons during the whole movie period are shown. *, *P*<0.05.

For the standard set of the movies, the population of G neurons showed significant increases in the normalized activity during the movie period, compared to that for the no-contacts (NC) and mounting-movies (M), in both the two grooming-movies (G1, G2), and their averaged activities did not show any significant differences between the G1 and G2 movies (Fig. 6A, B) (G1, mean ± S.E., 1.28±0.12; G2, 1.35±0.14; M, 0.97±0.07; NC, 0.94±0.05; one-way ANOVA and post-hoc comparison with Tukey-Kramer test, *P*<0.05; G1 vs. NC, *P*<0.05; G2 vs. NC, *P*<0.05; G1 vs. M, *P*<0.05; G2 vs. M, *P*<0.05; M vs. NC, *P*>0.05; G1 vs. G2, *P*>0.05). Temporal profiles of the activity of G neurons appeared similar between two grooming-stimuli; an initial phasic activity followed by a sustained increase in the activity during the movie period (Fig. 6A). Next, to further examine the relationship between the temporal profiles of the population activity of G neurons and the contents of the grooming movies, we compared them (see Figure S3). The contents of the grooming movies included not only movements of the monkeys, but also appearances of the monkeys’ face in the movies. While grooming was continuously performed by the monkeys in the movies, the G neurons showed a sustained increase in the activity. We didn’t find a clear difference in the activity between grooming with the hands and the mouth (Figure S3). Also, the activity was not modulated by the appearances of the faces in the movie.

Similar to G neurons, the population of M neurons showed significant increases in the normalized activity, compared to that for NC and grooming (G) movies, in both mounting-movies (M1, M2), and their averaged activities were not significantly different between M1 and M2 movies (Fig. 6C, D) (M1, 1.67±0.22; M2, 1.52±0.17, G, 1.16±0.13; NC, 1.27±0.19; *P*<0.01; M1 vs. NC, *P*<0.05; M2 vs. NC, *P*<0.05; M1 vs. G, *P*<0.05; M2 vs. G, *P*<0.05; G vs. NC, *P*>0.05; M1 vs. M2, *P*>0.05). The activity of M neurons showed a peak for both M1 and M2 movies (M1, 1.40 s from the start of movie; M2, 2.40 s; Fig. 6C). By comparing temporal profiles of the population activity of the M neurons and the contents of the mounting movies (Figure S4), we found that the peaks of the activity appeared around the start of the double foot clasp position [49] (see the definition in Material and Methods, and also see movie clips of M1 and M2 movies in Fig. 1B) in mounting; the start of the double foot clasp position in mounting differed, being about 1.32 s for M1 and 2.48 s for M2 from the start of the movies. Also the increase in M neurons’ activity was strongly related to the start (i.e., the male pushed the female) of mounting (Figure S4). On the other hand, we didn’t find any relationships between M neurons’ activity and appearances of the faces in the movie.

**Figure 7:**
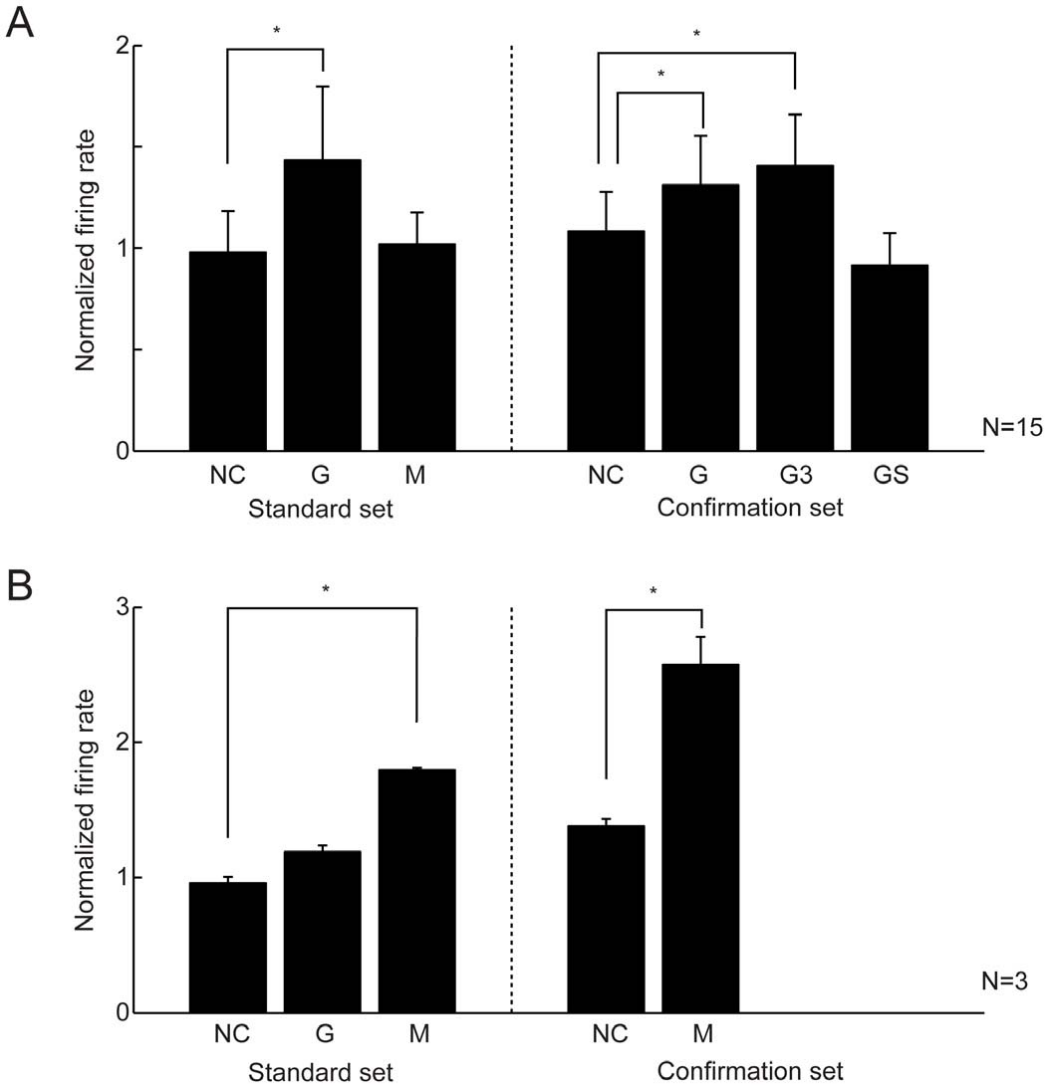
Population normalized-activities of G and M neurons tested with both the standard and the confirmation sets of movies. Mean normalized activities (mean ± S.E.) of (A) G (n  = 15) and (B) M (n = 3) neurons during the whole movie period are shown. NC, the averaged activity for the no-contacts movies. G, the averaged activity for the grooming movies conducted by two monkeys. M, the averaged activity for the mounting movies. G3, the averaged activity for the movies of grooming conducted by three monkeys. GS, the averaged activity for the movies of self-grooming of plural monkeys. *, *P*<0.05.

Furthermore, for the confirmation set of movies, the population of G and M neurons that tested with both the standard and the confirmation sets also showed significant increases in the normalized activity during the grooming and the mounting movies, respectively, compared to the activity for the other movies (Fig. 7). In addition, the population of G neurons showed significant increases in the activity for social-grooming conducted by two individuals (G) and three individuals (G3), but not for self-grooming (GS) (Figure 7A; G neurons (standard set): NC, 0.99±0.20; G, 1.43±0.36; M, 1.02±0.16; one-way ANOVA and post-hoc comparison with Tukey-Kramer test, *P*<0.05; G vs. NC, *P*<0.05; G vs. M, *P*<0.05; M vs. NC, *P*>0.05; G neurons (confirmation set): NC, 1.08±0.20; G, 1.310±0.24; G3, 1.40±0.25; GS, 0.91±0.16; *P*<0.05; G vs. NC, *P*<0.05; G3 vs. NC, *P*<0.05; GS vs. NC, *P*>0.05; G vs. G3, *P*>0.05; Figure 7B; M neurons (standard set): NC, 0.96±0.05; G, 1.19±0.05; M, 1.80±0.02; *P*<0.05; M vs. NC, *P*<0.05; M vs. G, *P*<0.05;G vs. NC, *P*>0.05; M neurons (confirmation set): NC, 1.38±0.06; M, 2.57±0.20; M vs. NC, *P*<0.05).

Thus, at the population level, G and M neurons showed differential activities in (social) grooming and mounting movies, respectively, in comparison with the others. Although temporal patterns of the population activity appeared to differ between G and M neurons, they appeared to be related to different time sequences of different social behaviors; the difference of temporal patterns of the activity would be attributed to the difference of temporal patterns of grooming and mounting.

### Quantitative Analyses for the Neuronal Categorization of Social Behaviors

**Figure 8:**
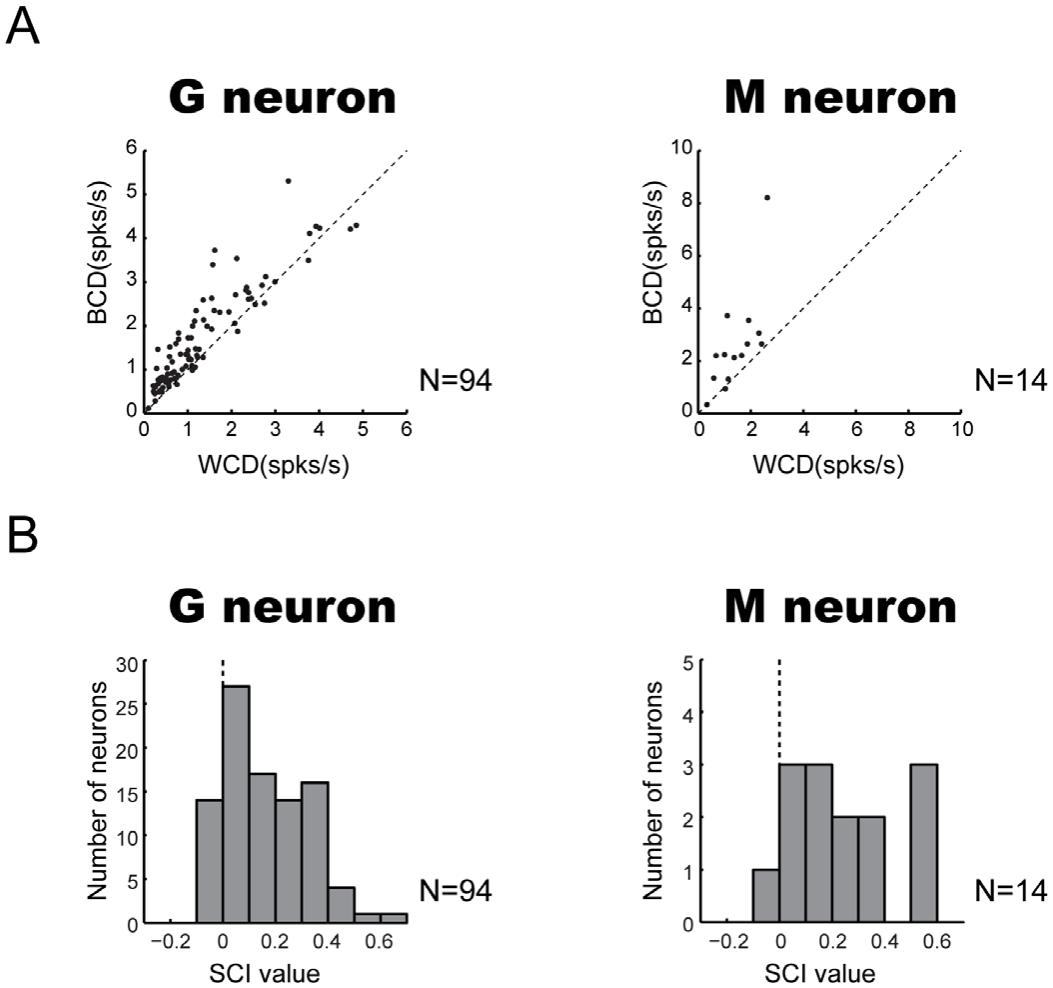
Quantitative analyses of the neuronal categorization of social behaviors. (A) For G and M neurons, average between-category differences (BCDs) and average within-category differences (WCDs) are shown. (B) For G and M neurons, social-behavior categorization index (SCI) values that were computed from BCD and WCD are shown. Positive values of indices indicate larger differences for movies in different categories and negative values indicate larger differences within each category.

Based on the above-mentioned findings, G and M neurons are likely to categorize the movies (i.e., for G neuron, grooming and non-grooming; for M neuron, mounting and non-mounting) at the population level (see Fig. 6B, D and Fig. 7). To examine this point quantitatively, we firstly evaluated whether individual neurons responded more similarly to movies within the same category than between different categories. For this quantitative evaluation, we computed an average within-category difference (WCD) and an average between-categories difference (BCD) for each type of neuron (see Materials and Methods). We found that WCDs were significantly smaller than BCDs for both G and M neurons (Fig. 8A; G neurons: WCD, mean ± S.E., 1.31±0.11 spikes/s; BCD, 1.69±0.11 spikes/s; paired *t*-test, *P*<0.05; M neurons: WCD, 1.44±0.19 spikes/s; BCD, 2.61±0.50 spikes/s; *P*<0.05). Thus, at the population level, the similarity of the activity (the spike rate in this case) was significantly stronger within the same category of movies than between different categories in both G and M neurons. Similar results were obtained by the same analysis for the confirmation set of movies (G neurons: WCD, mean ± S.E., 0.81±0.26 spikes/s; BCD, 1.08±0.29 spikes/s; paired *t*-test, *P*<0.05; M neurons: WCD, 0.93±0.52 spikes/s; BCD, 3.81±0.46 spikes/s, *P*<0.05).

To further quantify the categorization of social behaviors by G and M neurons, we calculated a social-behavior categorization index (SCI) (see Materials and Methods) that evaluates the “strength” of the neuronal categorization. Positive and larger values of this index indicate larger differences in the activity between two different categories of movies (i.e., stronger social-behavior categorization), and negative and smaller values indicate larger differences within the same category of movies. Distributions of SCI of G and M neurons were biased toward positive value (χ^2^ test, *P*<0.05 for G neuron; *P*<0.05 for M neuron), as shown in Fig. 8B. Also, medians of SCI values for both G and M neurons were greater than zero (Fig. 8B; G neurons: 0.15; M neurons: 0.19). These data indicate that the distribution of SCIs was shifted toward social-behavior categorization. Similar results were obtained by the same analysis for the confirmation set of movies (median of SCI values for G neurons: 0.18; M neurons: 0.74).

### Quantitative Analyses for the Neuronal Discrimination of Each Movie within Categories

The above-mentioned quantitative analyses indicate that G and M neurons categorize social movies. However, given the variability of the activity and SCI values across neurons, these neurons are likely to not only categorize movies but also discriminate each movie in the same category (e.g., Fig. 3B). To quantify and evaluate the neuronal discrimination of movies within the same category, we examined how many neurons showed a significantly different activity across different movies within the same category in the standard set. By this analysis, either G or M neurons were classified into two sub-types: “categorical” neurons that did not show a significantly different activity within the same category of movies (13/94 of G neuron; 3/14 of M neuron); and “discriminative” neurons that showed a significantly different activity among movies of the same category (81/94 of G neuron; 11/14 of M neuron). The majority were discriminative ones in both G and M neurons, and a large proportion of these neurons (86% for G neuron and 79% for M neuron) was statistically significant (χ^2^ test, *P*<0.05 for G neuron, *P*<0.05 for M neuron).

**Figure 9:**
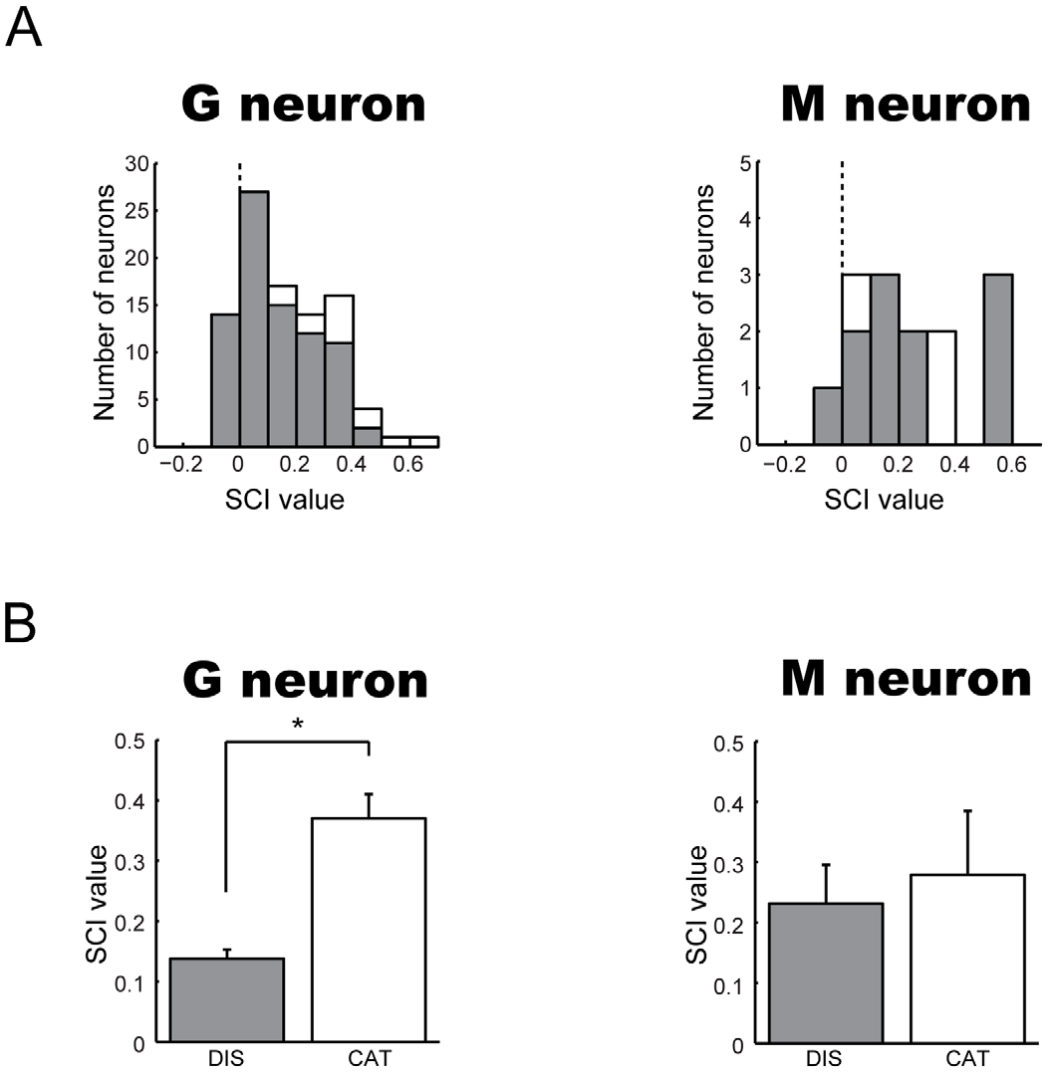
Comparison of the degree of the neuronal categorization in discriminative and categorical sub-types of G and M neurons. For G and M neurons, (A) distribution and (B) mean (± S.D.) of social-behavior categorization index (SCI) values that were computed by each sub-type of neurons are shown. White bar (Cat): categorical sub-type neuron, gray bar (Dis): discriminative sub-type neuron. *, *P*<0.05.

By their definition/criterion, these two sub-types of neurons (i.e., “discriminative” and “categorical” ones) must show different degrees of the neuronal categorization of social behaviors. To confirm this possibility, we compared the distribution of SCIs and averaged SCIs between these sub-types. The distribution of SCIs in categorical neurons shifted toward social-behavior categorization (i.e., greater than zero) than the distribution in discriminative neurons for G neurons (χ^2^ test, *P*<0.05), but not for M neurons (Fisher’s exact probability test, *P*>0.05) (Fig. 9A). Also, the average of SCIs in the categorical sub-type was significantly larger than that in the discriminative sub-type for G neurons (discriminative sub-type: mean ± S.E., 0.14±0.02; categorical sub-type: 0.37±0.04; *t*-test, *P*<0.05), but not for M neurons (discriminative sub-type: 0.23±0.06; categorical sub-type: 0.28±0.11; *P*>0.05) (Fig. 9B). However, a significant degree of the neuronal categorization was evident in both sub-types of G and M neurons, since 1) the proportions of neurons that had positive SCI values were significantly larger in both sub-types of G neurons and discriminative M neurons (Fig. 9A; discriminative G neurons, χ^2^ test, *P*<0.05; categorical G neurons, *P*<0.05; discriminative M neurons, *P*<0.05), 2) all SCIs of categorical M neurons were positive, although their bias was not significant (Fig. 9A; Fisher’s exact probability test, *P*>0.05), and 3) medians of SCIs for both subtypes of G and M neurons were greater than zero (Fig. 9A; discriminative G neurons, 0.10; categorical G neurons, 0.39; discriminative M neurons, 0.17; categorical M neurons, 0.38).

Thus, the majority of G and M neurons showed a significantly different activity within the same category of movies. However, the quantitative analysis revealed that even these neurons (i.e., discriminative ones) showed substantial degrees of the neuronal categorization of social behaviors. We found some differences between G and M neurons (e.g., the averaged SCI in the categorical sub-type was significantly larger than that in the discriminative sub-type for G neurons, but not M neurons). Such differences could be accounted for by the small number of each sub-type of M neurons (discriminative M neurons, n  = 11; categorical M neurons, n  = 3).

## Discussion

In the present study, we recorded the neuronal activity from the LPFC of monkeys during the presentations of movies of two types of social behaviors (grooming and mounting) and movies of plural monkeys without any eye- or body-contacts between them (no-contacts). We found that the movie-period activity of many LPFC neurons showed a significantly different activity for a particular type of social behavior in comparison with the others, and these neurons were classified into G (grooming) and M (mounting) neurons. In the present task, the subjects were not required to discriminate or memorize the movies. In addition, the subjects did not need to use any information/contents of the movies for a forthcoming decision/response (i.e., lever-release) like previous studies [51], [52], [53], [54], [55], [56], [57], [58]. Therefore, the movie-period activity of G and M neurons is suggested to be natural rather than a result of training of specific cognitive-functions.

We could not completely control various components of the movies (e.g., monkeys, background, temporal pattern of social behaviors, and length of movies). Nevertheless, we prefer to conclude that G and M neurons code/respond to grooming and mounting in the movie, respectively, because of the following two major findings: 1) each type of neuron showed a significant modulation during different movies with the same type of social behavior (i.e., grooming or mounting) but with different monkeys, backgrounds, temporal pattern of social behaviors and length of movies in the “standard set” at the individual neuron level and population level (Fig. 6B, D); and 2) exactly same results were obtained for the “confirmation set” of movies that is different from the standard set (Fig. 7). Furthermore, the significant modulation in the activity of G and M neurons was attributed to the overall pattern of movements of social behaviors rather than to small/local movements of faces/bodies and appearances of faces. This is because most G and M neurons showed a similar modulation of the activity, compared to the original movie, during the mosaic movies, but did not show it during the scrambled movies nor show any modulations according to the appearances of faces. The finding for the scrambled stimuli is similar to the findings for face-selective neurons in the prefrontal and temporal cortices; those face-selective neurons showed a decrement of the response to scrambled stimuli compared to original stimuli [26], [27], [59]. Thus, these findings suggest that G and M neurons encode grooming and mounting, respectively.

Although we prefer our interpretation of encoding of grooming and mounting by G and M neurons, respectively, we discuss two alternative possibilities. The first possibility is that the activity of G and M neurons might reflects other components (e.g., arousal, attention, and rewarding properties of social behaviors) that are associated with grooming and mounting rather than social behaviors themselves. Since we couldn’t completely dissociate encodings of social behaviors themselves and other possibilities and the neuronal activity in the LPFC shows attention- and reward-related modulations [60], [61], [62], [63], [64], we don’t exclude these possibilities. However, our indirect findings are unlikely to support that the activity of G and M neurons reflects arousal, attention, or reward value. For the most of the recording sessions, we didn’t find a significant difference in the reaction time across movies. Since different degrees of arousal, attention, or reward-value related to the movies are likely to lead different reaction times [58], [65], our result implies that monkeys didn’t differently arouse/attend to the movies nor did the activity of G and M neurons reflect different degrees of arousal, attention, or reward-value of the movies. Nevertheless, future studies should directly test whether/how much the modulation in the activity is explained by these factors.

The second possibility is that the activity of G and M neurons encodes non-social goal-directed behaviors, but not social behaviors. However, we conclude this possibility is unlikely based on following reasons. First, if G and M neurons encode non-social goal-directed behaviors independent of the type of behaviors, the neurons should respond to both the grooming and mounting movies. However, this was not the case. Instead, G and M neurons showed a modulation in the activity only during the grooming and mounting movies, respectively. How about the possibility that G and M neurons encode a specific type of non-social goal-directed behaviors? For G neurons, if the neurons encode non-social goal-directed behaviors related to grooming, similar neuronal responses should be observed during the self-grooming movies. However, most G neurons modulated their activities to social-grooming, but not self-grooming (Fig. 7B). This finding emphasizes the social nature of the activity of G neurons. Therefore, G neurons are likely to encode social grooming behaviors, but not non-social goal-directed behaviors related to grooming. For mounting, as far as we know, there aren’t mounting behaviors conducted by one individual and behavioral patterns of mounting are only observed during mounting behaviors (e.g., double foot clasping [49]). Therefore, goal-directed behaviors included in mounting are mostly social behaviors and strongly associated with mounting. Moreover, the peaks of M neurons’ activity were observed at the double foot clasping which is specific to mounting behaviors (Fig. 6C). Altogether, we conclude that M neurons are likely to encode mounting behaviors and/or social goal-directed behaviors included in mounting.

There was a proportional difference between G and M neurons, with a large number of G neurons (roughly seven times compared to M neurons). This may be related to some differences in character between grooming and mounting behaviors. Indeed, grooming plays many roles relative to mounting. Social grooming plays roles of 1) removing parasites [66], 2) reduction of tension [67], [68], 3) maintaining and reinforcing the sexual-bond/friendship [68], 4) post-conflict affiliate interactions [69], 5) increasing the probability that a groomee will tolerate the groomer [70] and 6) forming an alliance between a groomee and a groomer for agonistic conflict [70], [71]. In contrast, mounting plays roles of 1) mating behavior [49], [72], 2) reducing agonistic tendencies [73] and 3) greeting function to reinforce dominance relationships [74]. Furthermore, the length of time spent on grooming in social behaviors is longer than that on mounting [75]. Thus, these differences in character (i.e., roles, the length of time spending) between grooming and mounting might induce the proportional difference between G and M neurons.

Grooming and mounting behaviors in the field vary depending on several components, such as individuals, place and situation, but can be categorized by human observers. As an indirect evidence for the categorization in monkeys, it is suggested that monkeys recognize types of social behaviors, independent of the particular individuals involved: e.g., a monkey was more likely to threaten an opponent if one of its own close relatives and one of its opponent’s close relatives had recently been involved in a fight [2]. As for the social vocalization, monkeys categorize food-related calls in the absence of training [53], [56], and the neuronal categorization for such calls/vocalizations has been demonstrated in the LPFC [22], [24]. Therefore, it is likely that monkeys categorize social behaviors and have a neuronal mechanism for the categorization of social behaviors. As the activity of G and M neurons were considered to be related to such a neuronal categorization at the population level (Fig. 6B, D and Fig. 7), we examined whether the neuronal coding of G and M neurons is categorical by introducing quantitative measurements of WCD, BCD and SCI (Fig. 8). With these examinations, we obtained substantial evidences that the neuronal coding of G and M neurons is categorical at the neuronal population level. Furthermore, the large proportion of G and M neurons showed a significantly different activity within the same category of movies (i.e., discriminative sub-type neurons), but even such neurons showed a significant degree of the neuronal categorization (Fig. 9). The remaining small proportion of G and M neurons did not show a significantly different activity within the same category of movies (i.e., categorical sub-type neurons), and such neurons also showed a significant degree of the neuronal categorization (Fig. 9). Taken together, these findings suggest that LPFC neurons process abstract categorical information of social behaviors as well as discriminative information within their category; a hierarchical processing for social information (abstract categorical level – exact discriminative level within the same category) might progress within the LPFC.

Our findings and interpretation of the categorization and the discrimination within the category in LPFC neurons are consistent with some recent findings which suggest that LPFC neurons process categorical information and discriminative information of vocalizations. For example, the neuronal categorization in the LPFC has been demonstrated for vocalizations conveying information about food quality [22] or foods and non-foods [24]. Furthermore, other studies on the response of LPFC neurons to vocalizations showed that the LPFC neurons’ response is based on the acoustic morphology [30], suggesting the discriminative nature of the LPFC activity for vocalizations. Together with our findings and previous ones, it is likely that ecologically significant information in the field, in particular social information, is categorized and discriminated by LPFC neurons. This discrimination and categorization of social information by LPFC neurons may be adaptive for monkeys when they are required to simultaneously classify social information into more than one dimension. In fact, it has been demonstrated that monkeys simultaneously classify others according to both the individual rank and the kinship in the field [76], [77].

In this study, we provided an evidence for the processing of categorical and discriminative information of social behaviors by LPFC neurons, even when the information is not required for controlling behaviors. Recent studies suggest that other brain areas are also involved in the processing of social information or the production of emotional/social behaviors: e.g., evaluation of social movies and monitoring others’ action in the anterior cingulate cortex [9], [10], [11], generation of social gestures in the insula [12], social gaze following and social-context dependent neuronal modulation in the lateral intraparietal area [13], [14]. Therefore, it appears that each brain areas play a partially-overlapped, but differential role for the processing of social information. Here, what’s the characteristic role of the LPFC? Given the category sensitivity of neurons in the inferior temporal cortex, which sends direct projections to the LPFC, was weaker than that of the LPFC neurons [21], the LPFC may play a key role for the neuronal categorization of the social behaviors. More importantly, since the LPFC of monkeys plays a central role in decision makings and flexible control of goal-directed behaviors in a given situation [78], [79], [80], [81], [82], [83], a LPFC neuronal system associated with the categorization and discrimination of social behaviors may be linked with LPFC neuronal systems associated with decision makings and flexible control of goal-directed behaviors [83], thereby contributing to dynamic and flexible control of social interactions when they are required. Such a possible association/linkage between these two neuronal systems is worthy to be studied further for revealing the neuronal mechanism of social interactions/behaviors of group-living primates, including humans.

## Supporting Information

Figure S1 **Movie clips of original, mosaic, and scrambled movie stimuli of grooming, mounting, and no-contacts.**
(PDF)

Figure S2 **Lists for the contents of the movies.** Components related to the male (female) are shown in blue (red). Times indicate time from the movie start. For the grooming movies, we listed time points of grooming with the hands and grooming with the mouth. For the mounting movies, we listed time points that 1) the male pushed the female, 2) the female touched the ground, 3) the double foot clasp position started, 4) the male thrusted, 5) the male released the clasping of the female’s hind limbs, and 6) the female released her hands from the ground. Also we listed appearances of monkeys’ faces. We scored the appearance of face as 0, 0.5, or 1. If the face of the monkey was entirely observed, we scored it as 1. If the face was overlapped with another monkey’s body or the monkey showed the side face (i.e., only one eye was observed), we scored it as 0.5. If most parts of the face were not observed, we scored it as 0.
(PDF)

Figure S3 **Effects of contents of grooming movies on G neurons’ population activities.** Population activities of G neurons during the presentation of G1 and G2 movies (top row). Vertical lines indicate components of grooming movies (see insets below the panel). Male (female) face appearance scores are shown in middle (bottom) row.
(PDF)

Figure S4 **Effects of contents of mounting movies on M neurons’ population activities.** Population activities of M neurons during the presentation of M1 and M2 movies (top row). Vertical lines indicate contents of mounting movies (see insets below the panel). Male (female) face appearance scores are shown in middle (bottom) row.
(PDF)

Video S1 **Grooming movies.**
(MPG)

Video S2 **Mounting movies.**
(MPG)

Video S3 **No-contacts movies.**
(MPG)
