# Supplementary material for: Neuronal Categorization and Discrimination of Social Behaviors in Primate Prefrontal Cortex
Source: PLoS One. 2012 Dec 28;7(12):e52610. doi: 10.1371/journal.pone.0052610 (PMC3532303; doi:10.1371/journal.pone.0052610)
Supplement: Figure S1 — Movie clips of original, mosaic, and scrambled movie stimuli of grooming, mounting, and no-contacts. (PDF) [file pone.0052610.s001.pdf]

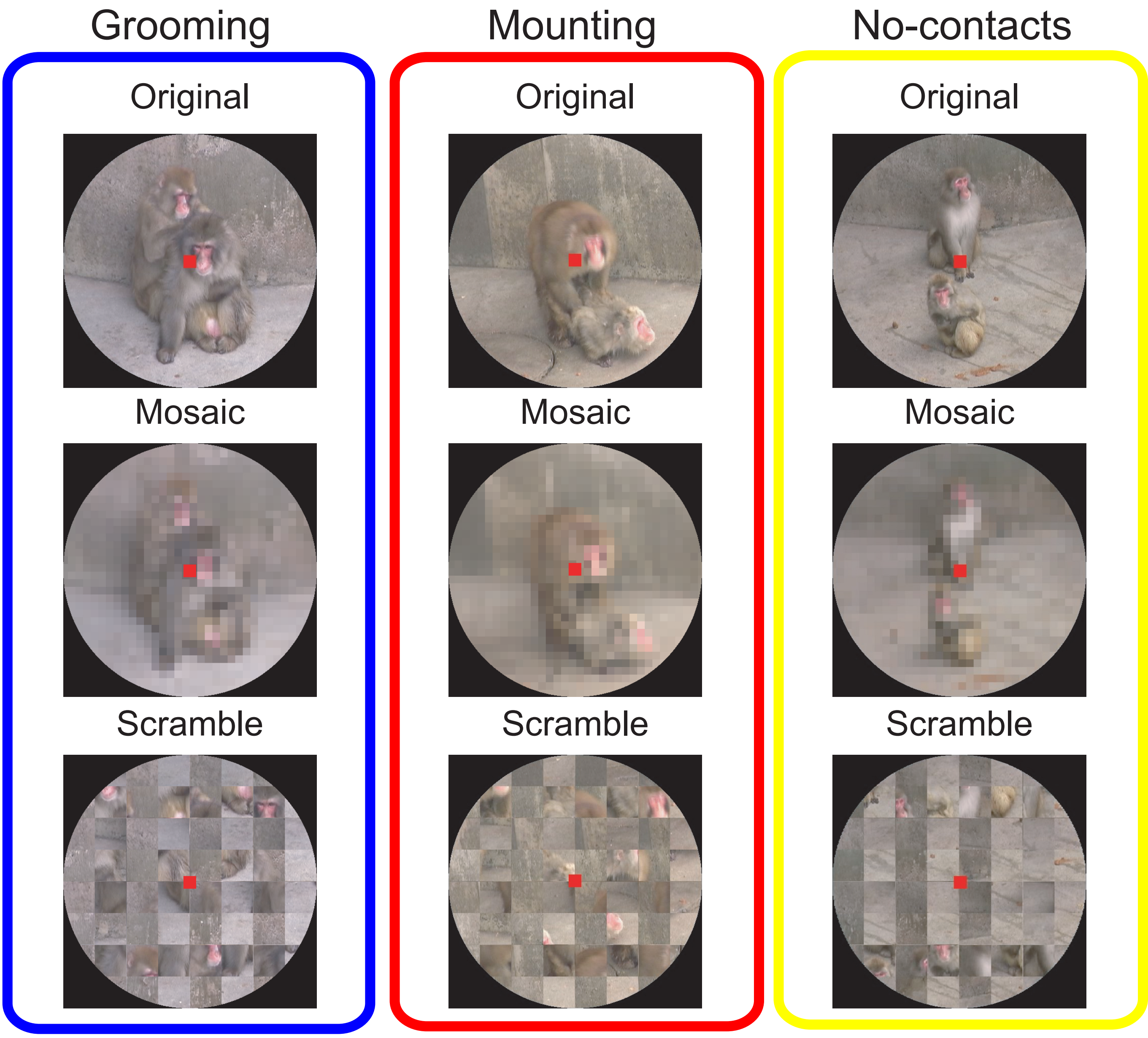
Grooming
Mounting
No-contacts
Original
Original
Original
Mosaic
Mosaic
Mosaic
Scramble
Scramble
Scramble

Figure S1.
