# Supplementary material for: Neuronal Categorization and Discrimination of Social Behaviors in Primate Prefrontal Cortex
Source: PLoS One. 2012 Dec 28;7(12):e52610. doi: 10.1371/journal.pone.0052610 (PMC3532303; doi:10.1371/journal.pone.0052610)
Supplement: Figure S2 — Lists for the contents of the movies. Components related to the male (female) are shown in blue (red). Times indicate time from the movie start. For the grooming movies, we listed time points of grooming with the hands and grooming with the mouth. For the mounting movies, we listed time points that 1) the male pushed the female, 2) the female touched the ground, 3) the double foot clasp position started, 4) the male thrusted, 5) the male released the clasping of the female’s hind limbs, and 6) the female released her hands from the ground. Also we listed appearances of monkeys’ faces. We scored the appearance of face as 0, 0.5, or 1. If the face of the monkey was entirely observed, we scored it as 1. If the face was overlapped with another monkey’s body or the monkey showed the side face (i.e., only one eye was observed), we scored it as 0.5. If most parts of the face were not observed, we scored it as 0. (PDF) [file pone.0052610.s002.pdf]

G1

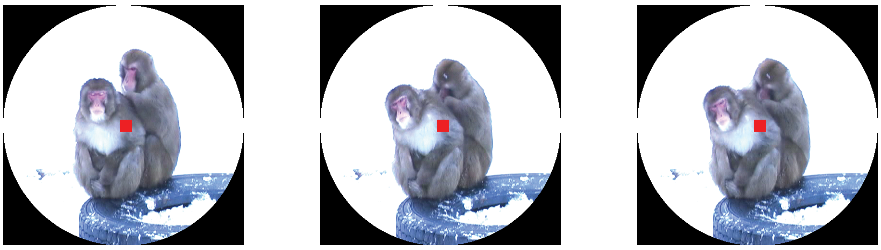

a. Grooming with hands. Face: 1, 1. (0 s)

b. Grooming with hands. Face: 1, 0.5. (5.72 s)

c. Final image.

G2

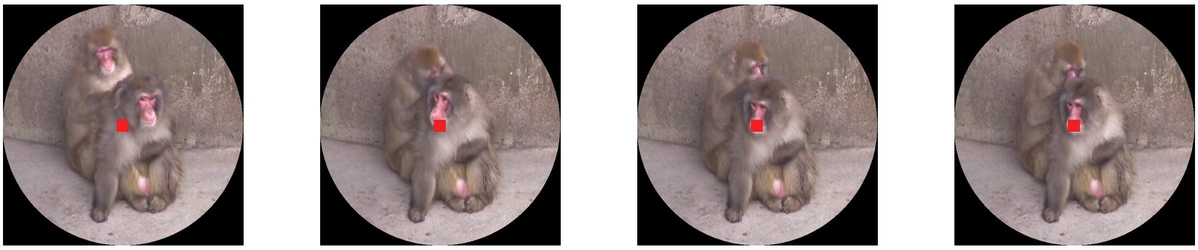

a. Grooming with hands. Face: 1, 1. (0 s)

b. Grooming with mouth. Face: 1, 0.5. (2.96 s)

c. End of mouth grooming. Face: 1, 0.5. (5.32 s)

d. Final image.

M1

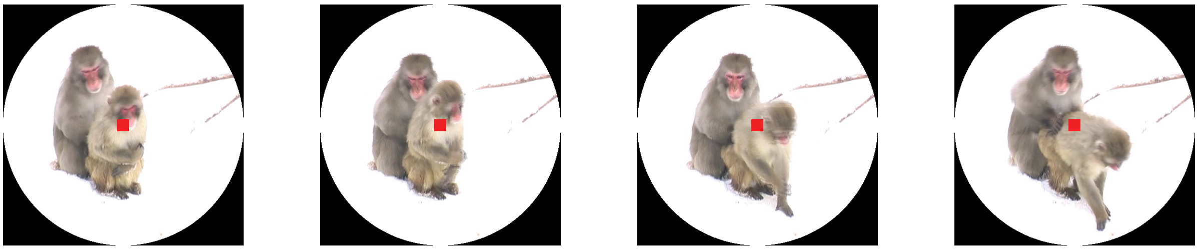

a. Movie start. Face: 1, 1. (0 s)

b. Pushing female. Face: 1, 0.5. (0.36 s)

c. Touching the ground (left hand). Face: 1, 0.5. (0.52 s)

d. Touching the ground (both hands). Face: 1, 0.5. (0.72 s)

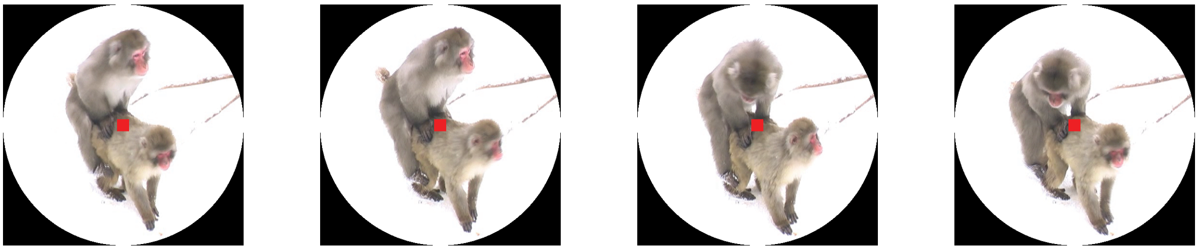

e. Clamping female's legs by male's legs. Face: 1, 1. (1.32 s)

f. Start of thrusting. Face: 1, 0.5. (1.64 s)

g. End of thrusting. Face: 0.5, 0.5. (3.52 s)

h. End of clamping legs. Moving left hand. Face: 0.5, 1. (3.92 s)

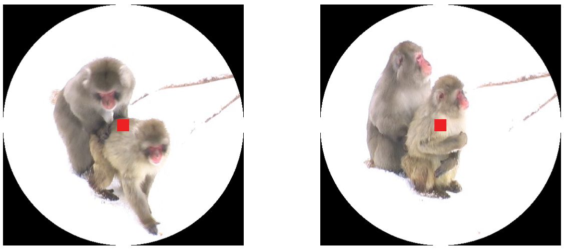

i. Moving right hand. Face: 1, 1. (4.2 s)

j. Final image.

Figure S2 (1/2).

M2

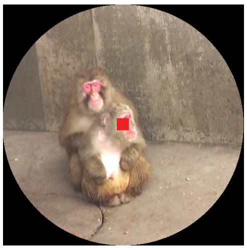

a. Movie start.
Face: 1, 1. (0 s)

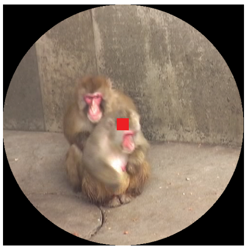

b. Pushing female.
Face: 1, 0.5. (1.32 s)

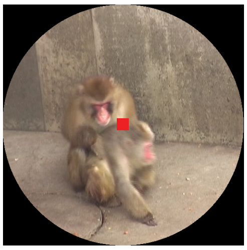

c. Touching the ground (right hand).
Face: 1, 0.5. (1.64 s)

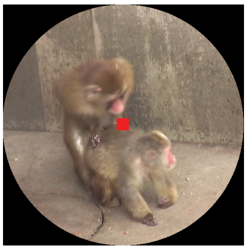

d. Touching the ground (both hands).
Face: 0.5, 0.5.
(1.84 s)

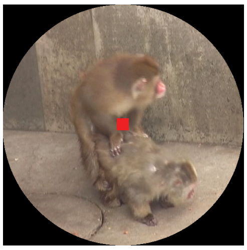

e. Clamping female's legs by male's legs.
Face: 0.5, 0.5.
(2.48 s)

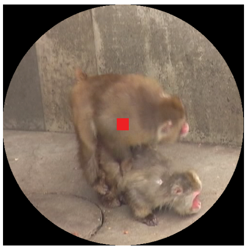

f. Start of thrusting.
Face: 0.5, 0.5.
(2.92 s)

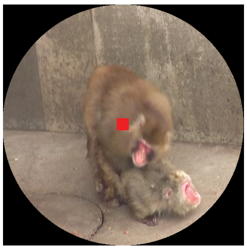

g. End of thrusting.
Face: 1, 0.5.
(4.72 s)

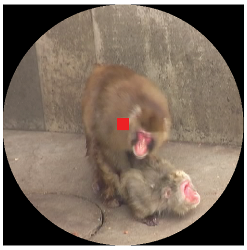

h. End of clamping legs. Face: 1, 0.5.
(4.8 s)

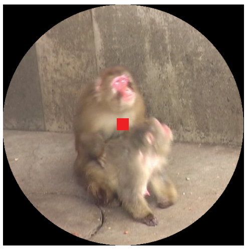

i. Moving left hand.
Face: 1, 0.5. (5.48 s)

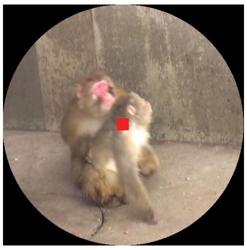

j. Moving right hand.
Face: 1, 0. (5.72 s)

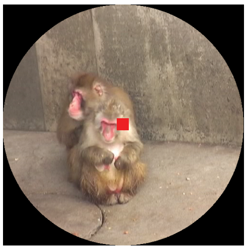

k. Final image.

NC1

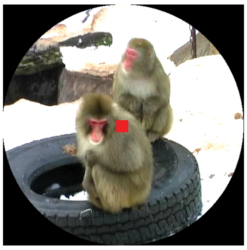

a. Movie start.
Face:1, 1. (0 s)

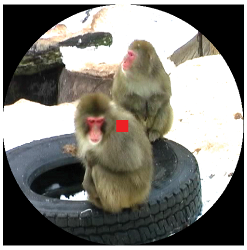

b. Final image.

NC2

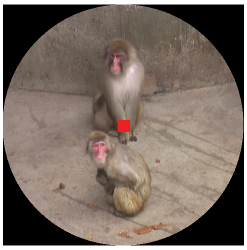

c. Movie start.
Face: 1, 1. (0 s)

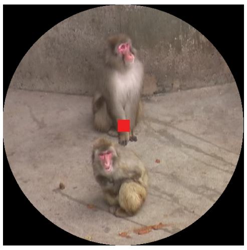

d. Final image.

Figure S2 (2/2).
