# Supplementary figures and images for: Neuronal Categorization and Discrimination of Social Behaviors in Primate Prefrontal Cortex
Source: PLoS One. 2012 Dec 28;7(12):e52610. doi: 10.1371/journal.pone.0052610 (PMC3532303; doi:10.1371/journal.pone.0052610)

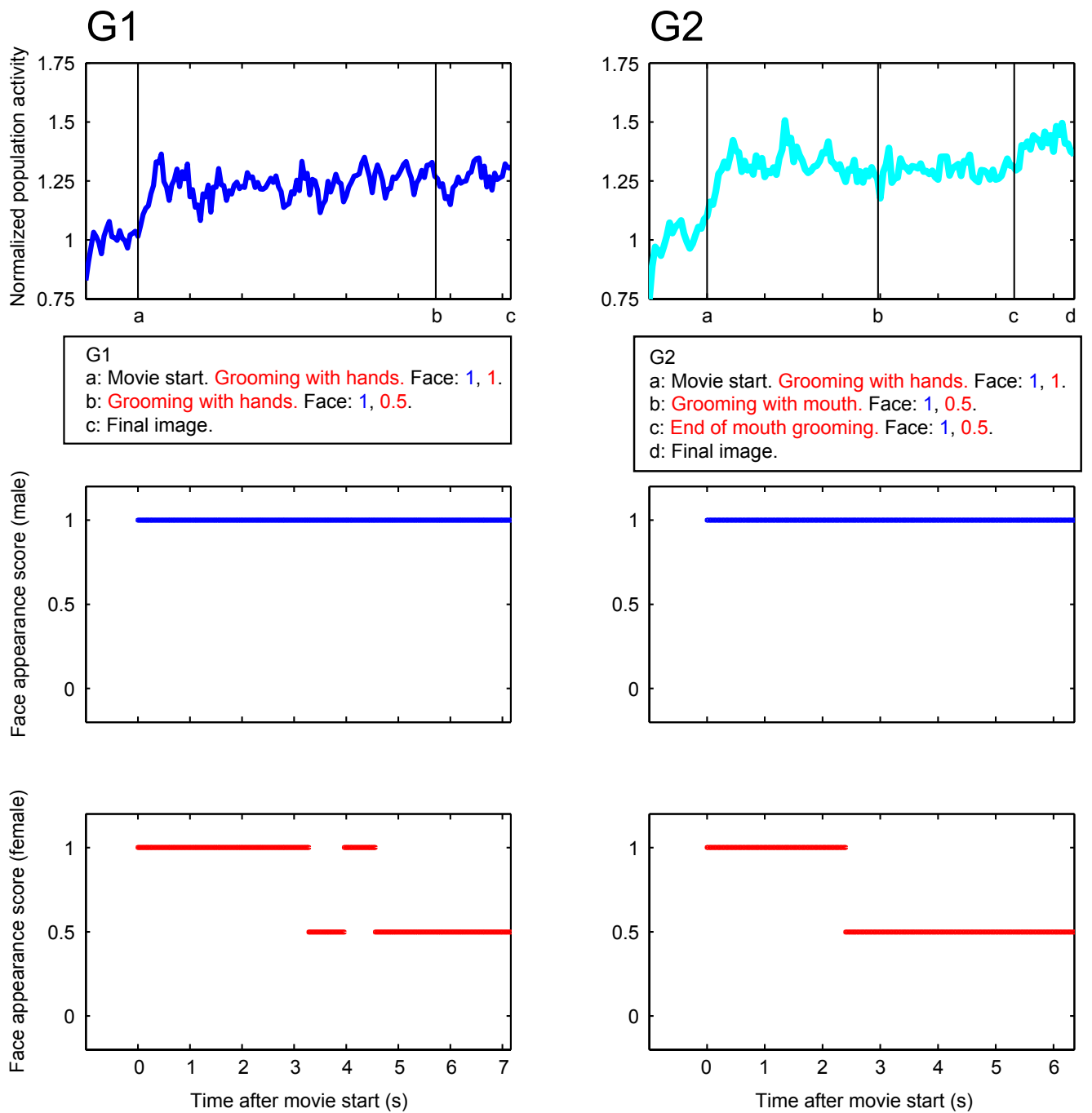

**Figure S3.**

Supplement: Figure S3 — Effects of contents of grooming movies on G neurons’ population activities. Population activities of G neurons during the presentation of G1 and G2 movies (top row). Vertical lines indicate components of grooming movies (see insets below the panel). Male (female) face appearance scores are shown in middle (bottom) row. (PDF) [file pone.0052610.s003.pdf]

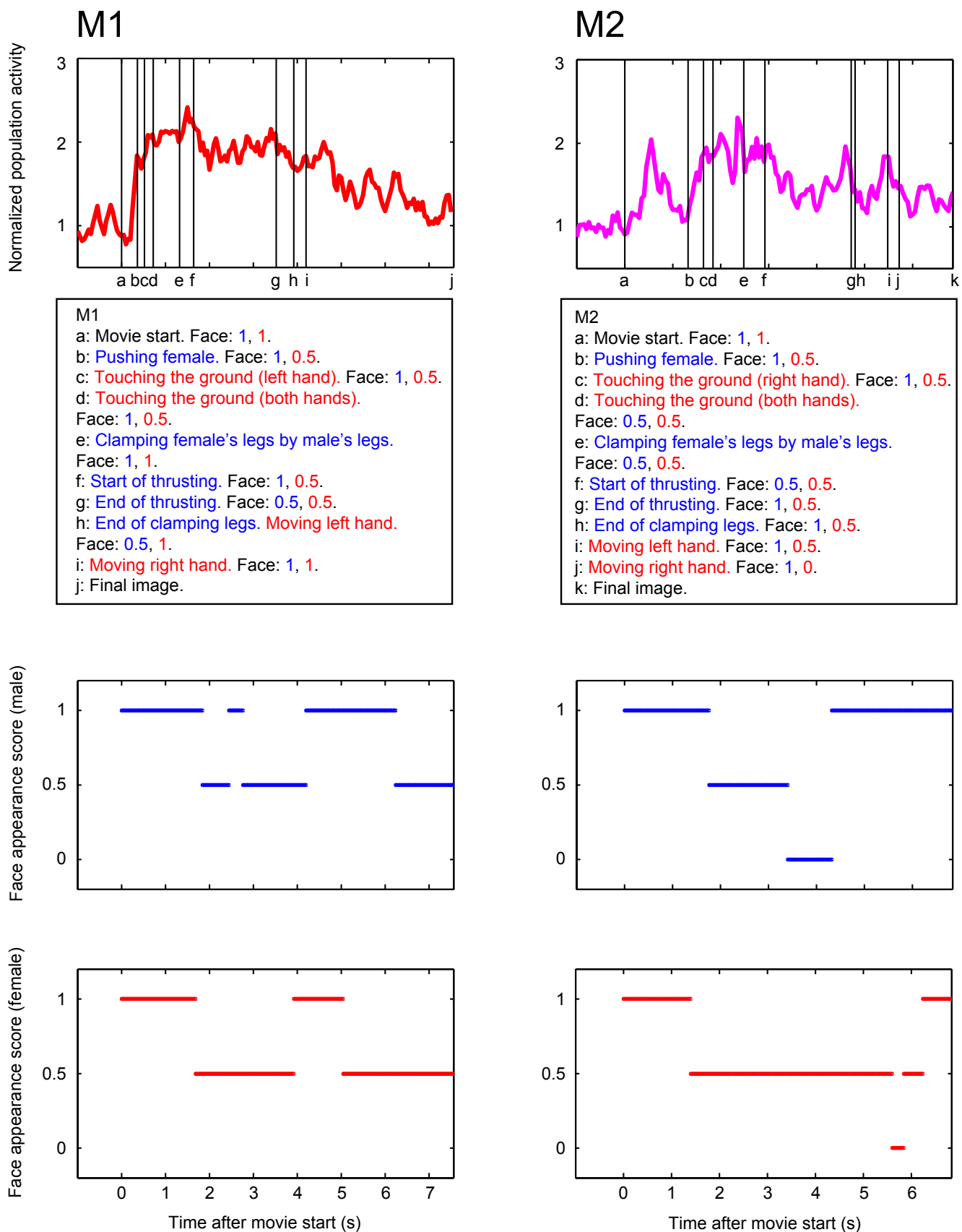

**Figure S4.**

Supplement: Figure S4 — Effects of contents of mounting movies on M neurons’ population activities. Population activities of M neurons during the presentation of M1 and M2 movies (top row). Vertical lines indicate contents of mounting movies (see insets below the panel). Male (female) face appearance scores are shown in middle (bottom) row. (PDF) [file pone.0052610.s004.pdf]
